# Enhancing gougerotin production by screening endogenous promoters for the transporter gene *gouM* in *Streptomyces albulus* CK-15

**DOI:** 10.3389/fmicb.2025.1719042

**Published:** 2026-01-13

**Authors:** Binghua Liu, Qianying Zhou, Ruixin Qiao, Kunping Zhou, Ning Zhang, Beibei Ge

**Affiliations:** 1 College of Agriculture and Forestry Science, Linyi University, Linyi, Shandong, China; 2 State Key Laboratory of Biology of Plant Diseases and Insect Pests, Institute of Plant Protection, Chinese Academy of Agricultural Sciences, Beijing, China

**Keywords:** endogenous promoter, transporter gene, gougerotin, *Streptomyces albulus*, high-yield strain

## Abstract

**Introduction:**

Gougerotin is a nucleoside antibiotic that exhibits strong inhibitory effects against bacteria, as well as activities against plant viruses and pathogenic fungi, making it highly valuable for development and application. However, its widespread use is limited by low production yield and long fermentation time. In the gougerotin biosynthetic gene cluster, the *gouM* gene is a transporter gene, and its encoded protein is responsible for transporting gougerotin to the extracellular space.

**Methods:**

To validate the impact of the *gouM* gene on gougerotin production, we generated a mutant strain by knocking out the *gouM* gene. To enhance the expression level of the *gouM* gene and thereby improve its ability to transport gougerotin, we screened endogenous promoters to drive the expression of the *gouM* gene through experiments including transcriptome sequencing, analysis, and measurement of mCherry fluorescence intensity.

**Results:**

The mutant strain *S. albulus* ΔgouM produced 508.34 mg/L of gougerotin, a 58.01% reduction compared to the wild-type strain (1212.74 mg/L). The results indicate that the transporter gene *gouM* significantly affects the yield of gougerotin.

We screened seven suitable endogenous promoters (PT1, PT2, PT3, PM3, PM4, PL2, and PL3). Each of these promoters was then used to drive the expression of the *gouM* gene. Among them, the gougerotin yields of strains with *gouM* driven by PT1 and PT2 promoters were 1451.11 mg/L and 1474.58 mg/L, which represented increases of 19.65% and 21.59%, respectively, compared to the wild-type strain.

**Discussion:**

This study demonstrates that using strong promoters to enhance the expression level of the transporter gene *gouM* can increase the yield of gougerotin, thereby providing a basis for subsequently using promoter engineering strategies to construct higher-yielding strains in the future.

## 1 Introduction

*Streptomyces*, a genus of Gram-positive bacteria, are widely found in natural habitats like soil and are known for producing diverse secondary metabolites with significant pharmaceutical, agricultural, and industrial applications (Donald et al., 2022; Jia et al., 2023). Under natural conditions, microorganisms produce relatively low yields of secondary metabolites to sustain their normal growth, which poses a major challenge for the industrial production of agricultural antibiotics (Alam et al., 2022). Therefore, improving antibiotic production in *Streptomyces* is of great importance for reducing costs and achieving industrial-scale manufacturing (Johnson et al., 2017).

In recent years, with the continuous development of molecular biology and bioinformatics, genetic engineering strategies (Martin and Liras, 2010; Liu et al., 2013), metabolic engineering strategies (Yu et al., 2019; Li et al., 2021), ribosome engineering strategies (Ochi et al., 2004), and synthetic biology strategies (Cummings et al., 2014) are currently the most commonly used approaches for constructing high-yielding strains of *Streptomyces* secondary metabolites. The implementation of these approaches has enhanced antibiotic biosynthesis in various *Streptomyces* strains. However, high-yield strains, as expected, are often not successfully screened. This is because the transport capacity of the transporter proteins responsible for exporting antibiotics from the intracellular to the extracellular space limits the extracellular antibiotic yield (Mukhopadhyay, 2015). Moreover, if antibiotics accumulate to excessive levels within cells due to untimely efflux, it imposes a burden on strain tolerance and ultimately limits final production (Naseri and Koffas, 2020).

Transporter proteins are encoded by transporter genes located either within or outside the antibiotic biosynthetic gene cluster. Their function is to secrete various substances, including antibiotics, out of the bacterial cell, thereby preventing excessive accumulation of antibiotics within the cell and avoiding self-inhibition (Martín et al., 2005; Liu et al., 2016). Based on the functions of transporter proteins, boosting the efflux capacity of encoded transporters through genetic modification is a key strategy to enhance transmembrane transport efficiency, protect cells from toxic byproducts, and increase the yield of diverse bacterial metabolites (Severi and Thomas, 2019; Steiger et al., 2019). In both the wild-type strain of the avermectin-producing bacteria and the constructed high-yield engineered strain, increasing the copy number of the transporter gene *avtAB* resulted in transporter gene-overexpressing strains with avermectin yields more than 2 times higher than those of the original strains (Qiu et al., 2011). In *Streptomyces bingchenggensis*, the expression of transporter genes TP2 and TP5 was regulated by screening suitable temporal promoters, thereby increasing milbemycin production by 36.9% and achieving the highest reported titer to date of 3,321 mg/L (Jin et al., 2020). Similarly, in *S. bingchenggensis*, different promoters were used to strongly express the transporter gene *miltAB2*. The excessive expression of *miltAB2*, driven by the strong promoter, imposed a significant metabolic burden, which in turn inhibited cellular growth. This indicated that when modifying transporter genes, it was necessary to balance the expression level of the transporter gene and cell growth. Therefore, Chu designed the TuPPE module with replaceable promoters and ribosome binding sites. By adapting different promoters for *miltAB2*, they optimized the expression level of the milbemycin transporter gene *miltAB2*. The resulting overexpression strain achieved a 24.2% increase in milbemycin production compared to the original strain (Chu et al., 2022).

Gougerotin is a nucleoside antibiotic first isolated in 1962 from *Streptomyces graminearus* (Iwasaki, 1962). It exhibits strong inhibitory effects against both Gram-positive and Gram-negative bacteria, along with antiviral, antifungal, anthelmintic, and acaricidal properties (Haneishi et al., 1974; Kondo et al., 1974; Lacal et al., 1980). The gougerotin biosynthetic gene cluster comprises a transcriptional regulator gene (*gouR*), a transporter gene (*gouM*), and thirteen biosynthetic genes (*gouA*–*gouL* and *gouN*). As a transporter gene within the cluster, the protein encoded by *gouM* primarily functions to transport gougerotin extracellularly (Niu et al., 2013; Jiang et al., 2013).

Recently, we discovered and isolated gougerotin from *Streptomyces albulus* CK-15. The gougerotin yield of *S. albulus* CK-15 is significantly higher than that of any previously reported wild-type strains, and the fermentation time was significantly shortened. To investigate the impact of the *gouM* gene on gougerotin production, we generated a *gouM* gene mutant strain through knockout experiments. The gougerotin yield of this mutant decreased by 58.01% compared to the wild-type strain. To enhance the expression of the *gouM* gene and consequently improve gougerotin transport, we performed promoter screening to identify optimal regulatory elements for its transcription. This effort resulted in two strains with increased gougerotin production, showing improvements of 19.65 and 21.59%, respectively, compared to the wild-type strain. This study demonstrates the efficacy of using strong promoters to upregulate the transporter gene *gouM* in boosting gougerotin yield, providing a foundation for developing higher-yielding strains in the future.

## 2 Materials and methods

### 2.1 Strains, plasmids, and growth conditions

All strains and plasmids used in this study were listed in Table 1. *S. albulus* CK-15 was obtained from the Wuyi Mountain in Fujian Province in China. The *Streptomyces* strains were cultured on mannitol soya flour MS (Liu et al., 2022) agar plates and cultivated in the malt extract-yeast extract-maltose (YEME) medium (Keijser et al., 2000) for liquid inoculation. *Escherichia coli* strains were grown in Luria-Bertani (LB) liquid broth or on LB agar plates. The fermentation medium used to produce gougerotin contained 2 g soybean flour, 2 g glucose, 3 g corn flour, 300 mg CaCO_3_, and 400 mg (NH_4_)_2_SO_4_ per 100 mL. *E. coli* strain ET12567/pUZ8002 was used for intergeneric conjugation from *E. coli* to *S. albulus* CK-15. The pSET152 plasmid without the integrase and integration site attp carried the promoters into *Streptomyces*. The pKC1139 plasmid was used for gene knockout in *Streptomyces.*

**TABLE 1:** Strains and plasmids used in this study.

Strains and plasmids	Description	Source
*E. coli* DH*5*α strain	A host for plasmid cloning	Lab stock
*E. coli ET12567/pUZ8002*	Methylation-deficient *E. coli* for conjugation with the helper plasmid	Lab stock
*S. albulus* CK-15	Wild-type gougerotin producer	Lab stock
*S. albulus* ΔgouM	Knocking out *gouM* gene in *S. albulus* CK-15	This work
*S. albulus* PT1	The promoter PT1 drives the expression of the *gouM* gene.	This work
*S. albulus* PT2	The promoter PT2 drives the expression of the *gouM* gene.	This work
*S. albulus* PT3	The promoter PT3 drives the expression of the *gouM* gene.	This work
*S. albulus* PM3	The promoter PM3 drives the expression of the *gouM* gene.	This work
*S. albulus* PM4	The promoter PM4 drives the expression of the *gouM* gene.	This work
*S. albulus* PL2	The promoter PL2 drives the expression of the *gouM* gene.	This work
*S. albulus* PL3	The promoter PL3 drives the expression of the *gouM* gene.	This work
pKC1139	Plasmid used for gene-knockout in *Streptomyces*	Lab stock
pKC1139-gouM	*gouM* disruption plasmid based on pKC1139	This work
pSET152	Plasmid used for carrying the promoters and *mcherry* gene into *Streptomyces*	Lab stock
pSET1	pSET152 without the integrase and integration site attp carried the promoters into *Streptomyces*	This work
pSET-Pi-gouM	Pi represents different candidate promoters (PT1, PT2, PT3, PM3, PM4, PL2, PL3)	This work

### 2.2 Processing for transcriptome sequencing samples

The *S. albulus* CK-15 was cultivated in YEME medium at 28°C for 48 h under constant shaking at 220 rpm. The spore suspension was inoculated (2% v/v) into the gougerotin fermentation medium and then cultured in a shaking incubator at 28°C and 220 rpm for approximately 60 h. Five time points, namely 12, 24, 36, 50, and 60 h, were selected for sampling in triplicate based on the time curve of *S. albulus* CK-15 producing gougerotin and sent for transcriptome sequencing.

### 2.3 Transcriptome analysis and selection of candidate constitutive promoters

The gene transcriptional level was measured by Fragments Per Kilobase of transcript sequence per Millionsbase pairs sequenced (FPKM) (Trapnell et al., 2010). The transcriptional level of a gene reflects the promoter strength. Strong promoters are genes whose FPKM values were all in the top 300 at five time points. Genes that can be stably and abundantly expressed during the fermentation process. The moderate promoters were genes with the FPKM value of between 200 and 400 across all five time points. Weak promoters were genes with FPKM values ranging from 30 to 60 at all five time points.

### 2.4 Promoter cloning

The screened promoters were obtained by PCR amplification using the whole-genome DNA of S. albulus CK-15 as the template. The primers used for PCR were listed in Supplementary Table 1. The promoters were cloned and recombined into the plasmid pSET152-mCherry and controlled the transcriptional level of the *mCherry* gene. The original promoter of *gouM* was also inserted into the plasmid pSET152-mCherry (pSET152-PgouM-mCherry) as the control.

### 2.5 Assessing the strength of promoters

The plasmids pSET152-mCherry with the promoters (pSET152-Pi-mcherry, Pi represents different candidate promoters) were transferred into *S. albulus* CK-15 by conjugative transformation. The plasmid pSET152-PgouM-mCherry was also introduced into *S. albulus* CK-15 to serve as a control. The obtained strains were inoculated in YEME medium at 28 °C with shaking (220 rpm) for 48 h. The fermentation broth was added to a black microplate. The fluorescence value of mCherry was detected at an excitation wavelength of 580 nm. At the same time, the OD600 of the bacterial liquid was measured to eliminate the influence of different growth rates of the strains on the fluorescence value. The relative fluorescence intensity of the recombinant cells was evaluated by the fluorescence intensity per OD600 unit. All samples were tested in triplicate.

### 2.6 Total RNA extraction and qRT-PCR

The strains were collected from YEME medium or gougerotin fermentation medium, frozen in liquid nitrogen, and grounded into a powder. Sample collection and processing, as well as the qRT-PCR workflow setup, followed the same methods as in our previous study (Liu et al., 2018). The primers used for qRT-PCR were listed in Supplementary Table 1. All samples were tested in triplicate.

### 2.7 Construction of strains with candidate promoters driving the expression of the *gouM* gene

Using the plasmid pSET152 as a template, a linearized plasmid fragment lacking the integrase int and the integration site attp was obtained by PCR. The linear fragment was assembled into the circular plasmid pSET1 using recombination technology. The candidate promoter and *gouM* gene were ligated into the *Xba*I and *Bam*HI restriction enzyme sites in pSET1 to generate the plasmid pSET-Pi-gouM (Pi represents different candidate promoters). The primers used for PCR were listed in Supplementary Table 1. The pSET-Pi-gouM was transferred into *S. albulus* CK-15 by conjugative transformation. After single-crossover recombination, strains with the candidate promoter activating *gouM* were selected by apramycin resistance.

### 2.8 *gouM* gene deletion mutant

The upstream and downstream sequences of the *gouM* gene and the gentamicin resistance gene sequence were amplified by PCR. The above sequences were ligated into the pKC1139 plasmid using the Gibson assembly method (Gibson et al., 2008) to construct the pKC1139-gouM for the *gouM* knockout mutant. The constructed plasmid pKC1139-gouM was transferred into *S. albulus* CK-15 via conjugation, and double crossover mutants were selected as previously described (Liu et al., 2022). Primers TF/TR (Supplementary Table 1) were designed for the upstream and downstream regions of the *gouM* gene fragment. A 1,505 bp PCR product was amplified from the wild-type strain. In contrast, a 1,031 bp product was obtained from the mutant strain as a result of the target gene deletion and insertion of the gentamicin resistance gene.

### 2.9 Fermentation and gougerotin production assay

The *S. albulus* CK-15 was cultivated in YEME medium at 28 °C with shaking (220 rpm) for 48 h. The spore suspension was inoculated into gougerotin fermentation medium (2% v/v), followed by incubation in a shaking incubator (220 rpm) at 28 °C for approximately 72 h. The fermentation broth was filtered through filter paper and then further filtered using a 0.22 μm membrane filter. The production of gougerotin in the fermentation broth of each strain was detected by an Agilent 1,100 high-performance liquid chromatography (HPLC) system with a ZORBAXSB-C18 column (4.6 mm × 250 mm, i.d., 5 μm) at 25 °C. The mobile phase includes 92% H_2_O with 1% C_2_HCl_3_O_2_ and 8% methanol. The flow rate was set at 1 mL/min with the detection at 276 nm. Gougerotin had a retention time of 23 min in these conditions.

Assay of intracellular gougerotin production. Take 10 mL fermentation broth and centrifuge at 4°C and 4,000 rpm for 10 min. The resulting precipitate was washed twice with an equal volume of PBS to obtain the bacterial strain. Add 10 mL of pre-cooled extraction solution (acetonitrile: methanol: water = 20:30:50) to the bacterial cells, and subject them to ultrasonication on ice for 5–10 min. The mixture was then centrifuged at 12,000 rpm for 10–15 min, and the supernatant was collected. The crude lysate was filtered using a 0.22 μm membrane filter. Due to the low concentration of intracellular metabolites, LC-MS was selected for the detection of intracellular gougerotin content using a Waters LC/TQ system fitted with an ACQUITY UPLC BEH Amide column (130 Å, 1.7 μm, 2.1 mm × 100 mm) and a VanGuard column (130 Å, 1.7 μm, 2.1 mm × 5 mm). The mobile phase includes solution A, which was an aqueous solution of 5 mM ammonium acetate and B, which was acetonitrile at a flow rate of 0.3 mL/min. MS data were acquired using electrospray ionization in the positive mode. The precursor ion was m/z 444.1, with a fragmentor voltage of 100 V, a collision energy range of 13–58 eV, and an ion acceleration voltage of 4 V.

### 2.10 Measurement of mycelial biomass

Strains with candidate promoters driving the expression of the *gouM* gene and the wild strain were cultivated in YEME medium at 28 °C with shaking (220 rpm) for 48 h. Then the spores of each strain were inoculated into M3G medium (Ge et al., 2016), followed by incubation in a shaking incubator (220 rpm) at 28 °C for 72 h. After filtering the bacterial broth, it was dried at 90 °C until a constant weight was achieved, and the mycelial dry weight of each strain was recorded every 6 h.

### 2.11 Statistical analysis

Data analysis was performed using GraphPad Prism (version 8.0, GraphPad Software, United States), with analysis of variance (ANOVA) being conducted in SAS (version 9.1, United States).

## 3 Results

### 3.1 Analysis of the *gouM* gene sequence

The *gouM* gene has a length of 1,329 bp, encodes 442 amino acids, and the protein has a molecular weight of 46.87 kDa. It belongs to the Major Facilitator Superfamily. MFS is a large and diverse group of secondary transporters that includes uniporters, symporters, and antiporters (Reddy et al., 2012). MFS proteins facilitate the transport across cytoplasmic or internal membranes of a variety of substrates, including ions, sugar phosphates, drugs, neurotransmitters, nucleosides, amino acids, and peptides (Drew et al., 2021). GouM from S. *albulus* CK-15 shows 98.19% identity with the GouM (JQ307220.1) from *S. graminearus* (Supplementary Figure 1). GouM shared sequence identities of 80.32% with QRX95245 (CP070326.1) from *Streptomyces noursei* strain A-2-1, 75.79% with WSK16095 (CP108413.1) from *Streptomyces celluloflavus* strain NBC_01299, and 68.28% with WEH36588 (CP119145.1) from *Streptomyces* sp. AM 4-1-1. QRX95245, WSK16095, and WSK16095 were all predicted to belong to the MFS family transporters with unknown functions.

### 3.2 Influence of *gouM* deletion on gougerotin production

**FIGURE 1:**
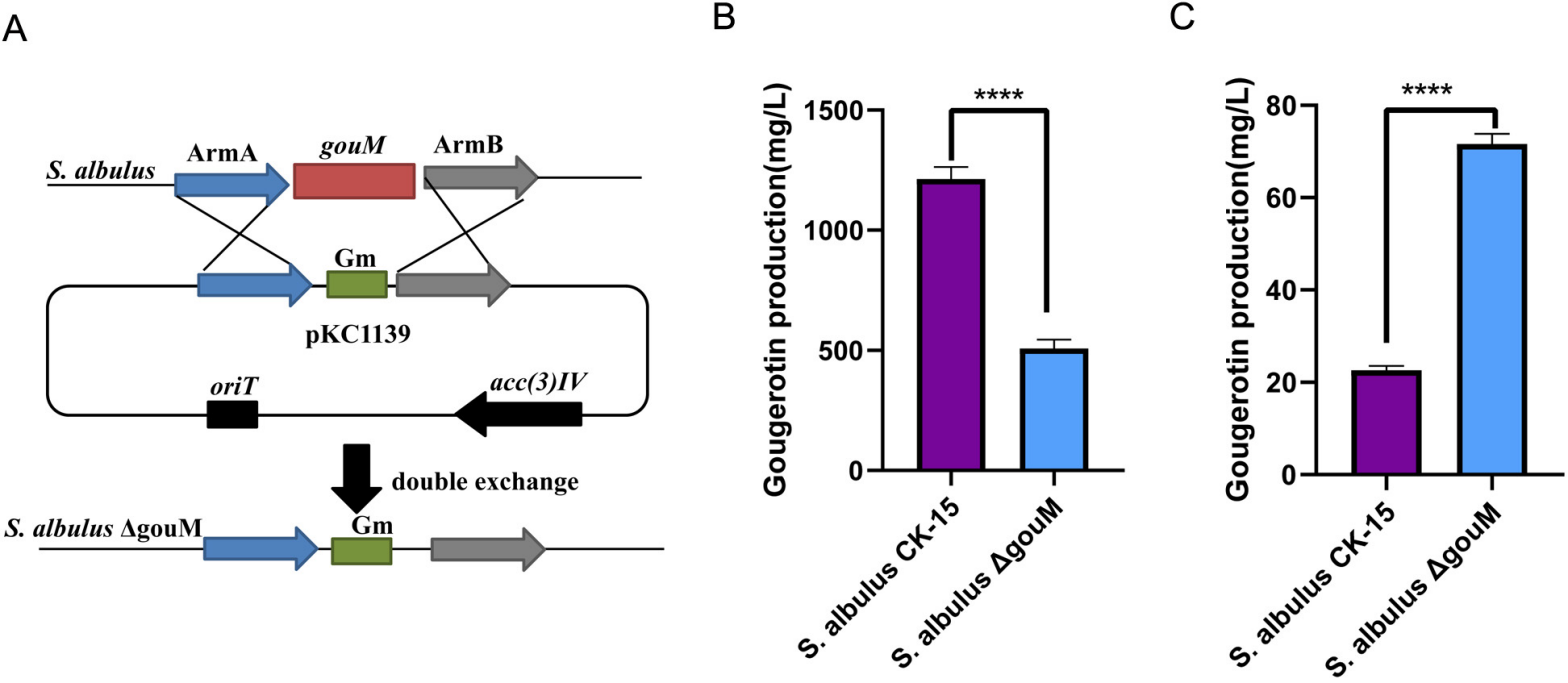
Influence of *gouM* deletion on gougerotin production. **(A)** Gene replacement of *gouM* in *S. albulus* CK-15. **(B)** Extracellular gougerotin production of WT and *S. albulus*ΔgouM. **(C)** Intracellular gougerotin content of WT and *S. albulus*ΔgouM. Error bars represent standard deviations of three biological replicates. **** means *p* < 0.0001.

To investigate the effect of the *gouM* gene on gougerotin production, we replaced the *gouM* gene with a gentamicin resistance gene via homologous double crossover (Figure 1A) and obtained the *gouM* mutant strain *S. albulus* ΔgouM through antibiotic selection (Supplementary Figure 2A). To assess the impact of *gouM* deletion, the wild-type and *S. albulus* ΔgouM strains were cultivated in gougerotin fermentation medium for 72 h, after which gougerotin yield in the broth was determined by HPLC analysis. The gougerotin content in the fermentation broth of each strain was quantified by applying the HPLC peak areas to the standard curve equation. The results showed that the gougerotin yield of the wild-type strain *S. albulus* CK-15 was 1212.74 mg/L (Figure 1B; Supplementary Figure 2C). The gougerotin yield of strain *S. albulus* ΔgouM was 508.34 mg/L (Figure 1B; Supplementary Figure 2D), which was 58.08% lower than that of the wild-type strain. The results showed a significant decrease in gougerotin production by the mutant strain. This reduction is attributed to the absence of the transporter protein, thereby preventing the efficient translocation of biosynthesized gougerotin into the fermentation broth. The intracellular gougerotin production was also detected by LC-MS. The yield of intracellular gougerotin in the wild-type strain was 22.67 mg/L (Figure 1C; Supplementary Figure 3B), while the yield in the *gouM* mutant strain was 71.66 mg/L (Figure 1C; Supplementary Figure 3C), which was three times that of the wild-type strain. This is because after the deletion of the *gouM* gene, gougerotin cannot be transported out of the cell in a timely manner, leading to its accumulation within the bacterium.

### 3.3 Expression of gougerotin biosynthesis gene in the *gouM* mutant strain

**FIGURE 2:**
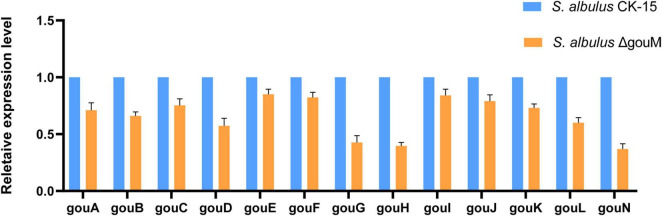
Expression level of gougerotin biosynthesis gene in *S. albulus* CK-15 and *S. albulus*ΔgouM. RNA was isolated from cells cultured in gougerotin fermentation medium for 72 h. To calculate relative gene expression, *S. albulus* CK-15 expression level was designated as 1. Error bars represent standard deviations of three biological replicates.

To investigate the impact of *gouM* gene deletion on the expression of other genes within the gougerotin biosynthetic gene cluster, we analyzed the transcription levels of *gouA* -*L* and *gouN* genes in *S. albulus* ΔgouM using qRT-PCR. The expression levels of these genes in the gougerotin biosynthetic gene cluster were all lower in the *gouM* mutant strain compared to the wild-type strain (Figure 2). In *S. albulus* ΔgouM, the expression levels of the *gouG*, *gouH*, and *gouN* genes are less than half of those in the wild-type strain. It is hypothesized that the deletion of the *gouM* gene resulted in the inability of the strain to promptly excrete intracellularly synthesized gougerotin. The high yield of intracellular gougerotin inhibited its biosynthesis.

### 3.4 Screening of constitutive promoters in *S. albulus* CK-15 via transcriptome rational analysis

Screening of constitutive promoters depended on the transcriptional level of genes whose expression profiles were stable. Three classes of promoters (strong, moderate, and weak) were selected according to the average FPKM values of their downstream genes. We amplified the 500 bp upstream and 100 bp downstream sequences of the putative translation start site (TSS) of genes as the constitutive promoters’ sequence (Jeong et al., 2016; Qin et al., 2021). After comprehensive screening, we successfully selected 8 strong, 5 moderate, and 5 weak promoters based on their expression intensities in Table 2.

**TABLE 2:** Select promoter genes with gradient strength based on FPKM values.

The strength of promoters	Promoters	Gene ID	Function	FPKM values
Strong promoters	PT1	gene26450	Glutaminase	1813.32
	PT2	gene15810	Succinyl-diaminopimelate desuccinylase	1474.97
	PT3	gene20000	Alkaline phosphatase	1231.66
	PT4	gene20735	Helix-turn-helix domain-containing protein	1223.28
	PT5	gene12200	Ubiquinol-cytochrome c reductase cytochrome b subunit	1095.82
	PT6	gene22580	Dipeptide ABC transporter ATP-binding protein, FtsH	979.75
	PT7	gene12885	MBL fold metallo-hydrolase	914.77
	PT8	gene28375	Phosphoribosyltransferase, IspG	855.19
Moderate promoters	PM1	gene17850	LysR family transcriptional regulator	280.162
	PM2	gene27235	Hypothetical protein	268.84
	PM3	gene28470	Hypothetical protein	263.77
	PM4	gene18915	DUF6230 family protein	261.96
	PM5	gene36125	Condensation protein	255.07
Weak promoters	PL1	gene12240	SDR family oxidoreductase	39.85
	PL2	gene34450	DUF4118 domain-containing protein	39.513
	PL3	gene18595	VOC family protein	39.07
	PL4	gene25935	SigE family RNA polymerase sigma factor	39.02
	PL5	Gene28990	NAD(P)H-binding protein	36.55

### 3.5 Characterization of the cloned promoters via mCherry fluorescence intensity

The mCherry fluorescence intensity correlates with the promoter strength. To assess the strength of the selected constitutive promoters, we measured their activity by quantifying mCherry fluorescence intensity. All candidate promoters were cloned upstream of the mCherry reporter gene, subsequently ligated into the pSET152 to construct recombinant plasmids were listed in Supplementary Table 2, and then individually transformed into *S. albulus* CK-15 for functional characterization.

Furthermore, we introduced a triple stop codon (TAAGTAGGTGA) at the 3′-end of each promoter sequence. This design effectively terminates any potential translation events, thereby eliminating possible interference from the coupled ribosome-binding site (RBS) and downstream transcription start site (TSS) (Qin et al., 2023). As a result, it not only ensures the proper functioning of the selected promoters at the transcriptional level but also prevents potential effects from the coupled RBS at the translational level (Li et al., 2018).

**FIGURE 3:**
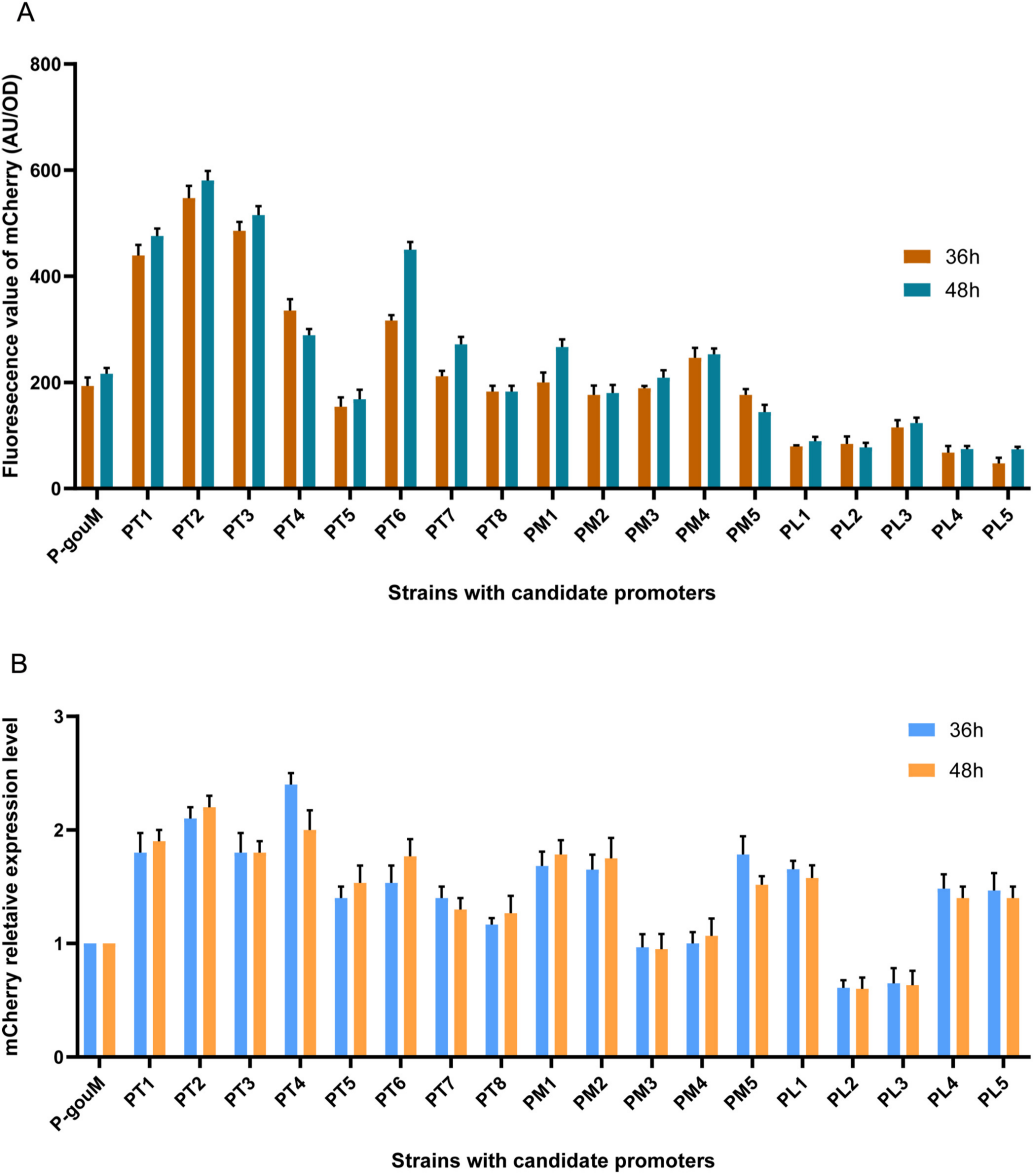
Characterization of the candidate promoters via mCherry fluorescence and qRT-PCR Analysis. **(A)** Relative fluorescence intensity of mCherry driven by the promoters was measured at 36 and 48 h. **(B)** Transcriptional levels of the *mcherry* gene under the control of different promoters were quantified at 36 and 48 h. RNA was isolated from cells cultured in YEME medium. Error bars represent standard deviations of three biological replicates.

Fluorescence intensity measurements were performed on samples collected at two time points (36 and 48 h) during the culture period. As shown in Figure 3A, the mCherry fluorescence intensity exhibited a broad activity range spanning (37.68–598.69). This result demonstrates that these promoters possess distinct capabilities for driving mCherry gene expression. The fluorescence values of the strains corresponding to promoters PT4, PT6, PT7, PM1, PM5, PL1, and PL5 showed excessive variation after 36 and 48 h of cultivation, indicating unstable expression of the mCherry under the control of these promoters. The expression levels of PT5 and PT8 are too low, which is inconsistent with the transcriptomic data analysis results. Therefore, the aforementioned promoters were not suitable for proceeding to the next step of initiating the expression of the *gouM* gene.

### 3.6 The transcriptional levels of *mcherry* driven by candidate promoters via qRT-PCR analysis

To validate the mCherry fluorescence analysis results, we further quantified the transcriptional levels of *mcherry* driven by candidate promoters using qRT-PCR (Figure 3B). The expression levels of the *mcherry* gene in strains corresponding to promoters PT4, PT5, PT6, and PM5 showed significant differences between 36 and 48 h. The expression levels of the *mcherry* gene in strains corresponding to promoters PM1, PM2, PL1, PL4, and PL5 are inconsistent with the fluorescence measurement results.

Based on comprehensive transcriptome data analysis, fluorescence value determination, and qRT-PCR analysis, we selected three strong promoters (PT1, PT2, and PT3), two moderate promoters (PM3 and PM4), and two weak promoters (PL2 and PL3) with stable expression to construct the strains in which candidate promoters were used to initiate the expression of the *gouM* gene.

### 3.7 Gougerotin production of strains with the candidate promoters activating *gouM*

**FIGURE 4:**
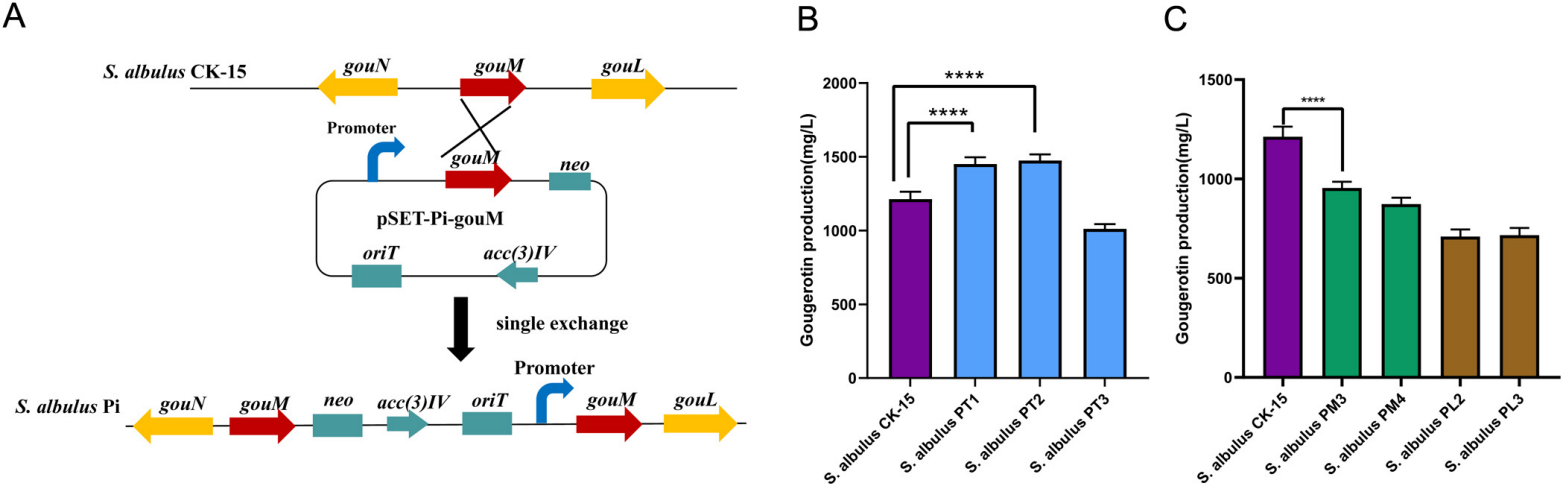
Gougerotin production of strains with the candidate promoters activating *gouM.* **(A)** The candidate promoter activating *gouM* gene. **(B)** Gougerotin production of strains with the promoter PT1, PT2 and PT3 activating *gouM.* **(C)** Gougerotin production of strains with the promoter PM3, PM42, PL2, and PL3 activating *gouM.* Error bars represent standard deviations of three biological replicates. **** means *p* < 0.0001.

Strains with the candidate promoter activating *gouM* via homologous single crossover (Figure 4A) and obtained the strains through apramycin resistance (Supplementary Figure 4). The seven selected strains and the wild-type strain were subjected to fermentation culture, respectively. The fermentation broth of each strain was analyzed by HPLC (Supplementary Figure 5; Figures 4B,C). The results showed that the gougerotin yield of the wild-type strain *S. albulus* CK-15 was 1212.74 mg/L. The gougerotin yield of strain *S. albulus* PT1 with *gouM* driven by PT1, reached 1451.11 mg/L, representing a 19.65% increase compared to the wild-type strain. The gougerotin yield of strain *S. albulus* PT2, reached 1474.58 mg/L, representing a 21.59% increase compared to the wild-type strain. The gougerotin yield of strain *S. albulus* PT3 was 1011.90 mg/L, representing a 16.56% decrease compared to the wild-type strain. The gougerotin yield of strain *S. albulus* PM3 was 954.44 mg/L, representing a 21.31% decrease compared to the wild-type strain. The gougerotin yield of strain *S. albulus* PM4 was 873.37 mg/L, representing a 27.98% decrease compared to the wild-type strain. The gougerotin yield of strain *S. albulus* PL2 was 709.90 mg/L, representing a 41.46% decrease compared to the wild-type strain. The gougerotin yield of strain *S. albulus* PL3 was 716.84 mg/L, representing a 40.89% decrease compared to the wild-type strain. The experimental results showed that strong promoters PT1 and PT2 enhanced *gouM* expression and increased gougerotin production. In contrast, the use of strong promoter PT3, moderate promoters PM3 and PM4, or weak promoters PL2 and PL3 decreased the yield.

### 3.8 The transcriptional level of the *gouM* gene with constitutive promoters

**FIGURE 5:**
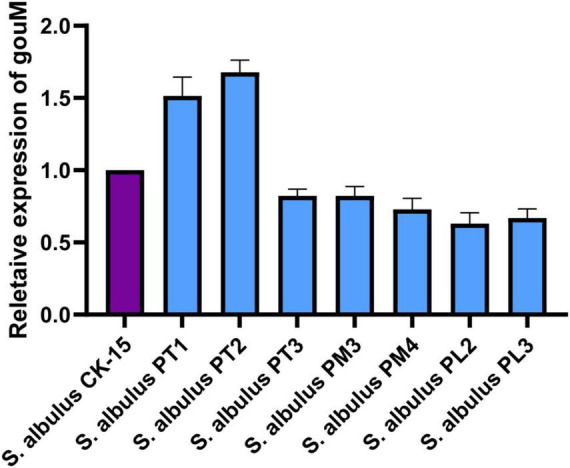
Expression level of *gouM* activated by candidate promoters. RNA was isolated from cells cultured in gougerotin fermentation medium for 72 h. To calculate relative gene expression *S. albulus* CK-15 expression level was designated as 1. Error bars represent standard deviations of three biological replicates.

The variation in gougerotin yield between the strains expressing the *gouM* gene under candidate promoters and the wild-type strain indicated that these promoters exert varying degrees of regulatory effects on the expression level of the *gouM* gene. To investigate the underlying mechanism, we analyzed the transcriptional level of *gouM* in the respective strains by qRT-PCR (Figure 5). The results indicated that the transcriptional levels of the *gouM* gene in strains with the strong promoters PT1 and PT2 were significantly higher than those in the wild-type strain. This trend was consistent with the changes in gougerotin production, suggesting that strong promoters effectively enhance the expression of the *gouM* gene, thereby improving the transport capacity for gougerotin. Conversely, the transcriptional levels of the *gouM* gene in strains utilizing the weak promoters PL2 and PL3 were significantly reduced, leading to a decrease in gougerotin production. Additionally, strains with the strong promoter PT3 or the moderate promoters PM3 and PM4 showed lower *gouM* transcription levels than the wild-type, and correspondingly, their gougerotin yields were also reduced.

### 3.9 The biomass of strains with candidate promoter activating *gouM*

**FIGURE 6:**
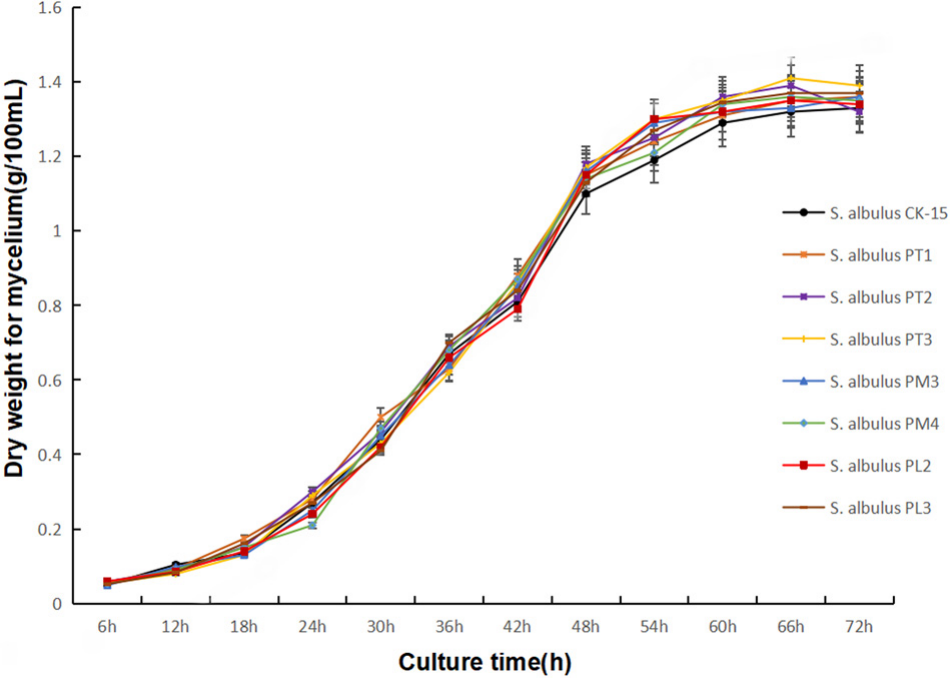
Result of mycelial biomass measurement. Biomass of strains with the candidate promoters activating *gouM* during culture for 72 h in M3G medium. Error bars represent standard deviations of three biological replicates.

To investigate whether the *gouM* gene affects the growth rate of *S. albulus* CK-15, the biomass of strains during the growth process was determined. Compared to the wild-type strain, the seven constructed strains showed no significant differences in biomass or growth status, indicating that the expression level of the gougerotin transporter gene *gouM* does not affect strain growth (Figure 6). These results indicated that the variation in gougerotin production among the engineered strains was not due to differences in growth rates but rather to the expression level of the *gouM* gene. The *gouM* expression level influences gougerotin yield by modulating the transport capacity of its encoded transporter.

## 4 Discussion

The promoter is a DNA sequence recognized, bound, and activated by RNA polymerase to initiate transcription. Its activity directly influences the level of gene expression, making it a key element in gene regulation and a decisive factor in the initiation of transcription (Liu et al., 2024). Since the initial proposal of promoter engineering in 2005, establishing extensive promoter libraries to quantitatively and accurately evaluate gene expression has gradually become a common and effective strategy in the field of metabolic engineering (Alper et al., 2005). Promoter engineering primarily regulates metabolic networks by controlling transcriptional levels. Integrating translational levels and post-translational modifications to optimize the enzyme expression process can further enhance the precision of gene expression levels and increase the yield of metabolic products (Jin et al., 2019). Rational tuning of gene expression through promoter selection is critical to enhancing microbial production of target compounds (Zhou et al., 2017). The microbial genome contains a variety of endogenous promoters, which are regarded as a vast resource for promoter engineering and metabolic engineering (Jin et al., 2019). Various methods, such as error-prone PCR (Gruet et al., 2012), transcriptome data analysis (Xu et al., 2019), and rational promoter design (Siegl et al., 2013), have been employed to establish promoter libraries in bacteria and fungi. The strength of a promoter can be significantly influenced by the genetic background of the host strain. Therefore, identifying endogenous strong promoters within the host strain may be more effective for optimizing biosynthetic pathways than using heterologous or artificial promoters (Zhang et al., 2021). Heterologous promoters have a weaker ability to be recognized and bound by RNA polymerase, leading to drawbacks such as low transformation efficiency and even interference with gene expression. In contrast, endogenous strong promoters may be key to enhancing transcription initiation efficiency and achieving high-level, stable expression of target genes (Okamoto et al., 2010). Only a limited number of promoters have been confirmed to drive heterologous gene expression in *Streptomyces*, including the constitutive promoters ermE*p (Bibb et al., 1985), SF14p (Labes et al., 1997), and kasO*p (Wang et al., 2013). The wild-type strain *S. albulus* CK15 yields a substantially higher gougerotin titer compared to other gougerotin-producing wild-type strains. Therefore, screening for suitable strong promoters in this strain to drive the expression of the *gouM* gene is likely a more feasible strategy for obtaining a high-yield strain. Furthermore, the promoters identified in this process can be utilized in subsequent studies on *S. albulus* CK-15, thereby expanding the toolkit of available promoters for future research.

In this study, based on transcriptome sequencing analysis, we identified and screened a set of 18 endogenous promoters with varying strengths, which were categorized as strong, moderate, and weak. Through experiments such as mCherry fluorescence value measurement, seven promoters were identified to drive the *gouM* gene, respectively. Among them, the PT1 and PT2 promoters significantly enhanced the expression level of the *gouM* gene, thereby increasing the production of gougerotin (Figures 4B, 5). The other five promoters reduced the expression level of the *gouM* gene to varying degrees, and the production of gougerotin in the corresponding strains was significantly lower than that in the wild-type strain (Figure 4C). The strong promoter PT3, when driving the *mcherry* gene, resulted in higher expression levels and fluorescence values of mCherry compared to those driven by the native promoter of the *gouM* gene (Figures 3A,B). However, the use of the PT3 promoter to drive the *gouM* gene resulted in reduced expression (Figure 5). The reason is likely that the PT3 promoter, despite its apparent high FPKM in transcriptome data, exhibits a weaker binding affinity for the *gouM* gene, indicating that its regulatory effect is gene-specific and can vary depending on the target gene. The production of gougerotin varied when driven by different promoters, but no significant differences were observed in the growth phenotype or biomass of the strains during cultivation (Figure 6). This suggests that the transporter gene gouM has no observable impact on the growth of *S. albulus* CK-15.

The gougerotin biosynthetic gene cluster contains one transcriptional regulator gene (*gouR*), one transporter gene (*gouM*), and thirteen biosynthetic genes (*gouA-gouL* and *gouN*) (Niu et al., 2013). Among them, *gouA*, *gouF*, and *gouH* function together to catalyze the formation of 4-amino-CGA, the nucleoside skeleton. *gouB* is responsible for the amidation of the glycosidic carboxyl group. *gouC* and *gouD* are associated with the synthesis of the sarcosine moiety in the peptide part of gougerotin. *gouG*, *gouI*, and *gouL* are involved in the formation of D-serine, while the methyltransferase GouN catalyzes the N-methylation of glycine to form sarcosine. GouK contains an acetyl-CoA binding domain and is responsible for activating serine or glycine to form seryl-CoA or glycyl-CoA. GouJ, an N-acetyltransferase, catalyzes the condensation of the activated amino acid with the nucleoside moiety. The transcriptional regulator GouR inhibits the transcription of *gouB* but activates the transcription of *gouM*, thereby coordinating gougerotin biosynthesis and transport to regulate its production (Wei et al., 2014). As a transporter gene of the MFS family, the primary function of *gouM* is to export gougerotin out of the cell. After the knockout of the *gouM* gene, a small amount of gougerotin could still be detected in the fermentation broth (Figure 1B), which indicates the existence of other export systems besides the *gouM* gene. qRT-PCR results showed that although *gouM* was not involved in gougerotin biosynthesis, its knockout significantly reduced the expression levels of all biosynthetic genes in the gougerotin cluster. It is hypothesized that the deficiency of the transporter gene led to the intracellular accumulation of gougerotin, as it could not be efficiently exported, which in turn inhibited its own biosynthesis. However, this negative feedback mechanism was complex and cannot be accounted for solely by gene expression levels.

## Conclusion

In conclusion, activating the *gouM* gene with promoters of different strengths directly influenced its expression level and ultimately gougerotin production. In this study, we selected *S. albulus* PT1 and *S. albulus* PT2, which the production of gougerotin reached 1451.11 and 1474.58 mg/L (Figure 4B), respectively. That showed a 19.65 and 21.59% increase in gougerotin yield, respectively, compared to the wild-type strain. This study demonstrates the feasibility of enhancing gougerotin production by upregulating the expression of the *gouM* gene. This work provides a foundation for applying promoter engineering strategies to develop high-yielding gougerotin strains.

## Data Availability

The original contributions presented in this study are included in this article/Supplementary material, further inquiries can be directed to the corresponding authors.
