# Supplementary material for: Enhancing gougerotin production by screening endogenous promoters for the transporter gene gouM in Streptomyces albulus CK-15
Source: Front Microbiol. 2026 Jan 13;16:1719042. doi: 10.3389/fmicb.2025.1719042 (PMC12835213; doi:10.3389/fmicb.2025.1719042)
Supplement: Supplementary file 1 [file Data_Sheet_1.doc]

**Supplementary Material**

Supplementary Table S1. Primers used in this study

| Primer | Sequence (5ʹ–3ʹ) | Description |
| --- | --- | --- |
| M-L-F | CGACGGCCAGTGCCAAGCTGTCGCAGAGCAGGACGAACAC | Upstream region of *gouM* |
| M-L-R | TTTCCACGGTGTGCGTCTGCCGAAACCCGATGGAAT |
| M-R-F | AAATTGTCACAACGCCGCGTCACGGCACGGAGAAACAG | Downstream region of *gouM* |
| M-R-R | CTATGACATGATTACGAATTCTTCGTCGGGCGTGGTAGA |
| Gm-F | ATTCCATCGGGTTTCGGCAGACGCACACCGTGGAAA | Identification of the Gm-resistance gene |
| Gm-R | CTGTTTCTCCGTGCCGTGACGCGGCGTTGTGACAATTT |
| TF | ATTTCGCCGAGACCGTACAAG | Identification of *∆gouM* |
| TR | TTCTCGGA AACGGTCAACCAAG |
| Apra-F | GAGTGCAATGTCGTGCAATACGA | Identification of the Apra-resistance gene |
| Apra-R | GCATTCTTCGCATCCCGCCT |
| Goum-F | GTGAATTCCATCGGGTTTCG | *gouM* gene fragment |
| Goum-R | AGTGGGACACGCGTCGGTAC |
| mcherry-F | ATGGTGAGCAAGGGCGAAGA | *mcherry* gene fragment |
| mcherry-R | TCACTTGTACAGCTCGTCCA |
| P1-F | TGGGCTGCAGGTCGACTCTAGTACCTGGGCAACCGTCCGAT | Mcherry expression |
| P1-R | TCTTCGCCCTTGCTCACCATGGCTGCTCCATCTCCTCGTA |
| P2-F | TACGAGGAGATGGAGCAGCCATGGTGAGCAAGGGCGAAGA | Mcherry expression |
| P2-R | ATCGCGCGCGGCCGCGGATCTCACTTGTACAGCTCGTCCA |
| P3-F | TGGGCTGCAGGTCGACTCTAGGGAACAACTACGAGAAGGGC | Mcherry expression |
| P3-R | TCTTCGCCCTTGCTCACCATGTCATCCCTCACCGACGTTG |
| P4-R | CAACGTCGGTGAGGGATGACATGGTGAGCAAGGGCGAAGA | Mcherry expression |
| P4-R | ATCGCGCGCGGCCGCGGATCTCACTTGTACAGCTCGTCCA |
| P5-F | TGGGCTGCAGGTCGACTCTAGAACCTCGGCTCCGCCTACCT | Mcherry expression |
| P5-R | TCTTCGCCCTTGCTCACCATCGTGGAATTCCCTTCGTATGGT |
| P6-F | ACCATACGAAGGGAATTCCACGATGGTGAGCAAGGGCGAAGA | Mcherry expression |
| P6-R | ATCGCGCGCGGCCGCGGATCTCACTTGTACAGCTCGTCCA |
| P7-F | TGGGCTGCAGGTCGACTCTAGTTCGATCTGCAGTCGTTCCT | Mcherry expression |
| P7-R | TCTTCGCCCTTGCTCACCATCGACCTCGTCTTTCGTTGAC |
| P8-F | GTCAACGAAAGACGAGGTCGATGGTGAGCAAGGGCGAAGA | Mcherry expression |
| P8-R | ATCGCGCGCGGCCGCGGATCTCACTTGTACAGCTCGTCCA |
| P9-F | TGGGCTGCAGGTCGACTCTAGGCAGGCTGGTGTCGGCGTA | Mcherry expression |
| P9-R | TCTTCGCCCTTGCTCACCATCGTGCTGTGTCTCCTGTGCT |
| P10-F | AGCACAGGAGACACAGCACG ATGGTGAGCAAGGGCGAAGA | Mcherry expression |
| P10-R | ATCGCGCGCGGCCGCGGATCTCACTTGTACAGCTCGTCCA |
| P11-F | TGGGCTGCAGGTCGACTCTAGTACGGATTTCACGTTTGGCG | Mcherry expression |
| P11-R | TCTTCGCCCTTGCTCACCATGGGCTCGCAACCTCCCTGTA |
| P12-F | TACAGGGAGGTTGCGAGCCCATGGTGAGCAAGGGCGAAGA | Mcherry expression |
| P12-R | ATCGCGCGCGGCCGCGGATCTCACTTGTACAGCTCGTCCA |
| P13-F | TGGGCTGCAGGTCGACTCTAGGTTTTCCACGAGCCGCAGAT | Mcherry expression |
| P13-R | TCTTCGCCCTTGCTCACCATGCCCACCACCCTACTGACCG |
| P14-F | CGGTCAGTAGGGTGGTGGGCATGGTGAGCAAGGGCGAAGA | Mcherry expression |
| P14-R | ATCGCGCGCGGCCGCGGATCTCACTTGTACAGCTCGTCCA |
| P15-F | TGGGCTGCAGGTCGACTCTAGGTCTTTATGTGCGATAGTGCTCA | Mcherry expression |
| P15-R | TCTTCGCCCTTGCTCACCATCCCAGTATTGCGTGTCGGT |
| P16-F | ACCGACACGCAATACTGGGATGGTGAGCAAGGGCGAAGA | Mcherry expression |
| P16-R | ATCGCGCGCGGCCGCGGATCTCACTTGTACAGCTCGTCCA |
| P17-F | TGGGCTGCAGGTCGACTCTAGCCTCCCTCGTTGCCTACCT | Mcherry expression |
| P17-R | TCTTCGCCCTTGCTCACCATTCCTACTCCCGCTTTCGTAT |
| P18-F | ATACGAAAGCGGGAGTAGGAATGGTGAGCAAGGGCGAAGA | Mcherry expression |
| P18-R | ATCGCGCGCGGCCGCGGATCTCACTTGTACAGCTCGTCCA |
| pSET1-F | CCGTACTGACTCGAAAAGTTCGACAGCGTCTC | plasmid pSET1 |
| pSET1-R | AACTTTTCGAGTCAGTACGGGCCATAGAGGG |
| PgouM-F | GTTGTCGGCG TAGCGGTCCA | The promoter of gouM |
| PgouM-R | AACCGGAGGC ACCGGGGCG |
| PT1-F | TACCTGGGCAACCGTCCGAT | PT1 promoter gene fragment |
| PT1-R | GGCTGCTCCATCTCCTCGTA |
| PT2-F | GGAACAACTACGAGAAGGGC | PT2 promoter gene fragment |
| PT2-R | GTCATCCCTCACCGACGTTG |
| PT3-F | AACCTCGGCTCCGCCTACCT | PT3 promoter gene fragment |
| PT3-R | CGTGGAATTCCCTTCGTATGGT |
| PM3-F | TACGGATTTCACGTTTGGCG | PM3 promoter gene fragment |
| PM3-R | GGGCTCGCAACCTCCCTGTA |
| PM4-F | GCAGGCTGGTGTCGGCGTA | PM4 promoter gene fragment |
| PM4-R | CGTGCTGTGTCTCCTGTGCT |
| PL2-F | GTCTTTATGTGCGATAGTGCTCA | PL2 promoter gene fragment |
| PL2-R | CCCAGTATTGCGTGTCGGT |
| PL3-F | CCTCCCTCGTTGCCTACCT | PL3 promoter gene fragment |
| PL3-R | TCCTACTCCCGCTTTCGTAT |
| *hrdB*-F | TCCGTCTGGTGGTCTCCCTG | *hrdB* gene fragment |
| *hrdB*-R | GGAGAACTTGTAGCCCTTGGTG |
| qmcherry-F | AGGGCACCCA GACCGCCAAG | qRT-PCR analysis |
| qmcherry-R | CCAGCGGACATCCCGGACTA C |
| qgoum-F | CGAGCAGGGAGAGCAGGAGG | qRT-PCR analysis |
| qgoum-R | CGAGGTGGGCACGGTCATC |
| *gouA*-F | GGACCGGCCCGGGGGGCGGT | qRT-PCR analysis |
| *gouA*-R | GCAGGCACGGGCGCCGAATC |
| *gouB*-F | CCGTTGCCCACCATCACCA | qRT-PCR analysis |
| *gouB*-R | CGGGAGCCTTCAGCGACGA |
| *gouC*-F | TCGGGCAGCAGCAGGTCGCG | qRT-PCR analysis |
| *gouC*-R | AAGTAGTCGC TGCCCAGCAC |
| *gouD*-F | CGTTTCCTTGCGGTCGTTGG | qRT-PCR analysis |
| *gouD*-R | GCGTCATCATCCCCGCCTAC |
| gouE-F | CGACGACGAGTTCCTTGACC | qRT-PCR analysis |
| *gouE*-R | TCTTCGAGGTCCTGGTCCAC |
| *gouF*-F | AGCCGTCGGTTGACCTCCTG | qRT-PCR analysis |
| *gouF*-R | TGGAGCCAAGTCCTCATCTGC |
| *gouG*-F | AGGTGTCGCTCGGGTAGTCC | qRT-PCR analysis |
| *gouG*-R | GCATCCACGACAAACAACCC |
| *gouH*-F | TACAAGGCAAGGTTGGCCAC | qRT-PCR analysis |
| *gouH*-F | AGGTCAACAGTGCGGCGACG |
| *gouI*-F | TTGAGCCAGGTCAACTGCTC | qRT-PCR analysis |
| *gouI*-R | TCTGCAAGGTCGACACGATG |
| *gouJ*-F | CGACCAGGTGTCCGTAGAGC | qRT-PCR analysis |
| *gouJ*-R | GCGTGGCTCCTCCCAACTGC |
| *gouK*-F | ATCGCCTCGTCCACCAGC | qRT-PCR analysis |
| *gouK*-R | GGACCCCGACGGGACCT |
| *gouL*-F | ACGAACAGGGACAGCGAACC | qRT-PCR analysis |
| *gouL*-R | CACCCTCACCCCGCAGAC |
| *gouN*-F | CTCCAGTTGCTCTTGACGCC | qRT-PCR analysis |
| *gouN*-R | TACGCTCCCATTCTTCCACC |

Supplementary Table S2. Strains and plasmids used in this study

| Strains and plasmids | Description | Soure |
| --- | --- | --- |
| *S. albulus* mPT1 | The promoter PT1 drives the expression of the *mCherry* gene | This work |
| *S. albulus* mPT2 | The promoter PT2 drives the expression of the *mCherry* gene | This work |
| *S. albulus* mPT3 | The promoter PT3 drives the expression of the *mCherry* gene | This work |
| *S. albulus* mPT4 | The promoter PT4 drives the expression of the *mCherry* gene | This work |
| *S. albulus* mPT5 | The promoter PT5 drives the expression of the *mCherry* gene | This work |
| *S. albulus* mPT6 | The promoter PT6 drives the expression of the *mCherry* gene | This work |
| *S. albulus* mPT7 | The promoter PT7 drives the expression of the *mCherry* gene | This work |
| *S. albulus* mPT8 | The promoter PT8 drives the expression of the *mCherry* gene | This work |
| *S. albulus* mPM1 | The promoter PM1 drives the expression of the *mCherry* gene | This work |
| *S. albulus* mPM2 | The promoter PM2 drives the expression of the *mCherry* gene | This work |
| *S. albulus* mPM3 | The promoter PM3 drives the expression of the *mCherry* gene | This work |
| *S. albulus* mPM4 | The promoter PM4 drives the expression of the *mCherry* gene | This work |
| *S. albulus* mPM5 | The promoter PM5 drives the expression of the *mCherry* gene | This work |
| *S. albulus* mPL1 | The promoter PL1 drives the expression of the *mCherry* gene | This work |
| *S. albulus* mPL2 | The promoter PL2 drives the expression of the *mCherry* gene | This work |
| *S. albulus* mPL3 | The promoter PL3 drives the expression of the *mCherry* gene | This work |
| *S. albulus* mPL4 | The promoter PL4 drives the expression of the *mCherry* gene | This work |
| *S. albulus* mPL5 | The promoter PL5 drives the expression of the *mCherry* gene | This work |
| pSET152-Pi-mcherry | Pi represents different candidate promoters(PT1, PT2, PT3, PT4, PT5, PT6, PT7, PT8, PM1, PM2, PM3, PM4, PL1, PL2, PL3, PL4) | This work |

**
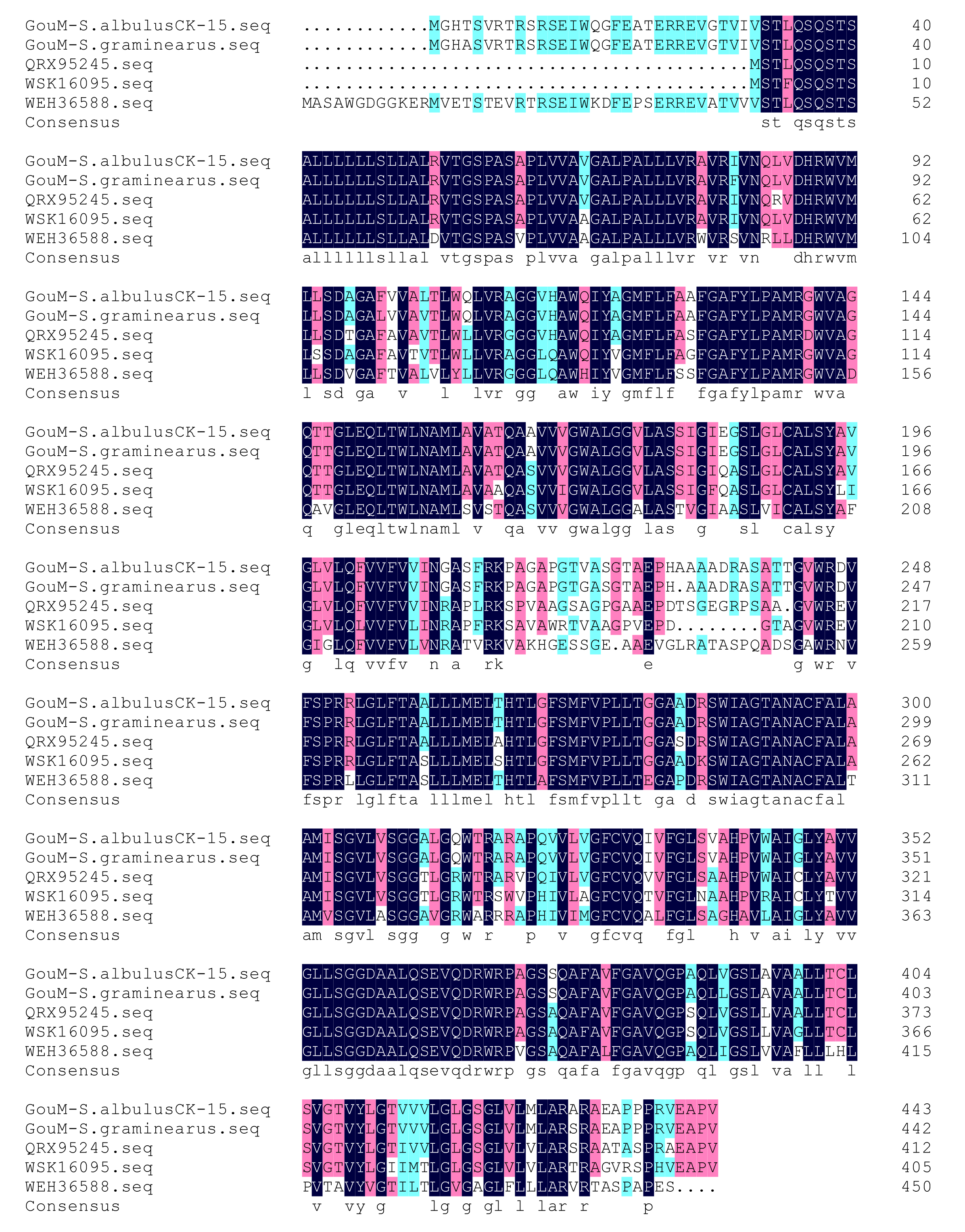
**

**Supplementary Figure 1.** Alignment of GouM protein with homologous proteins from other *Streptomyces* species. GouM-*S. albulus* CK-15, putative MFS family transporter from *S. albulus* CK-15 (GenBank accession number CP026094.1); GouM- *S. graminearus*, putative MFS family transporter from *S. graminearus* (GenBank accession number JQ307220.1); QRX95245, putative MFS family transporters from *S. noursei* strain A-2-1 (GenBank accession numberCP070326.1); WSK16095, putative MFS family transporters from *S. celluloflavus* strain (GenBank accession number CP108413.1); WEH36588, putative MFS family transporters from *Streptomyces sp*. AM 4-1-1(GenBank accession number CP119145.1).

**
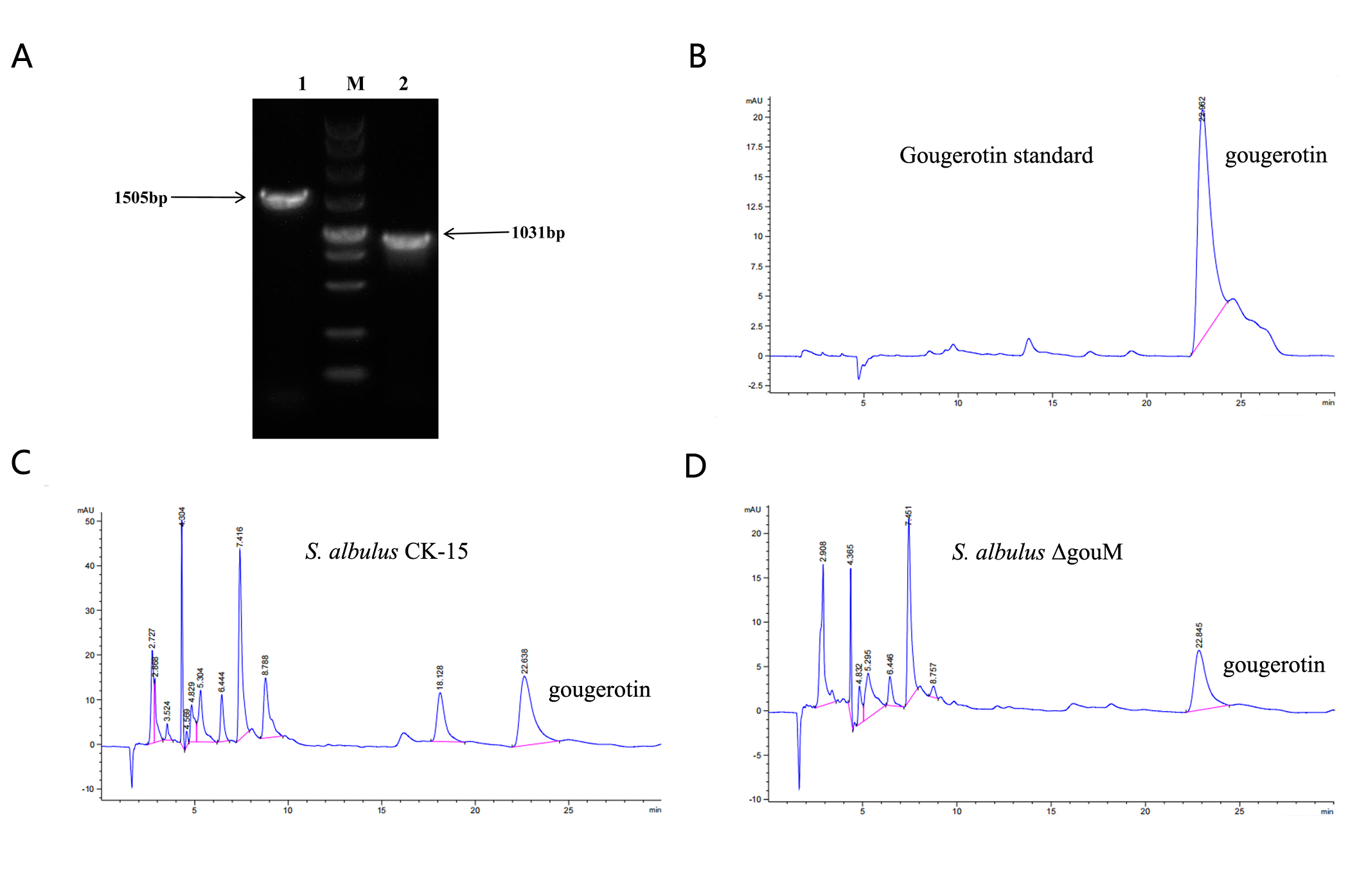
**

**Supplementary Figure 2. (A)** Confirmation of the constructed ∆gouM mutant by PCR. Lanes: M, BL 5,000-bp DNA ladder; 1, PCR verification with primers TF and TR using *S. albhlus* CK-15 genomic DNA as the template; 2, PCR verification with primers TF and TR using *S. albulus* ∆gouM genomic DNA as the template. **(B)** HPLC analysis of standard gougerotin. **(C)**HPLC analysis of gougerotin production levels in *S.albulus* CK-15. **(D)** HPLC analysis of gougerotin production levels in *S. albulus* ∆gouM*.*

**
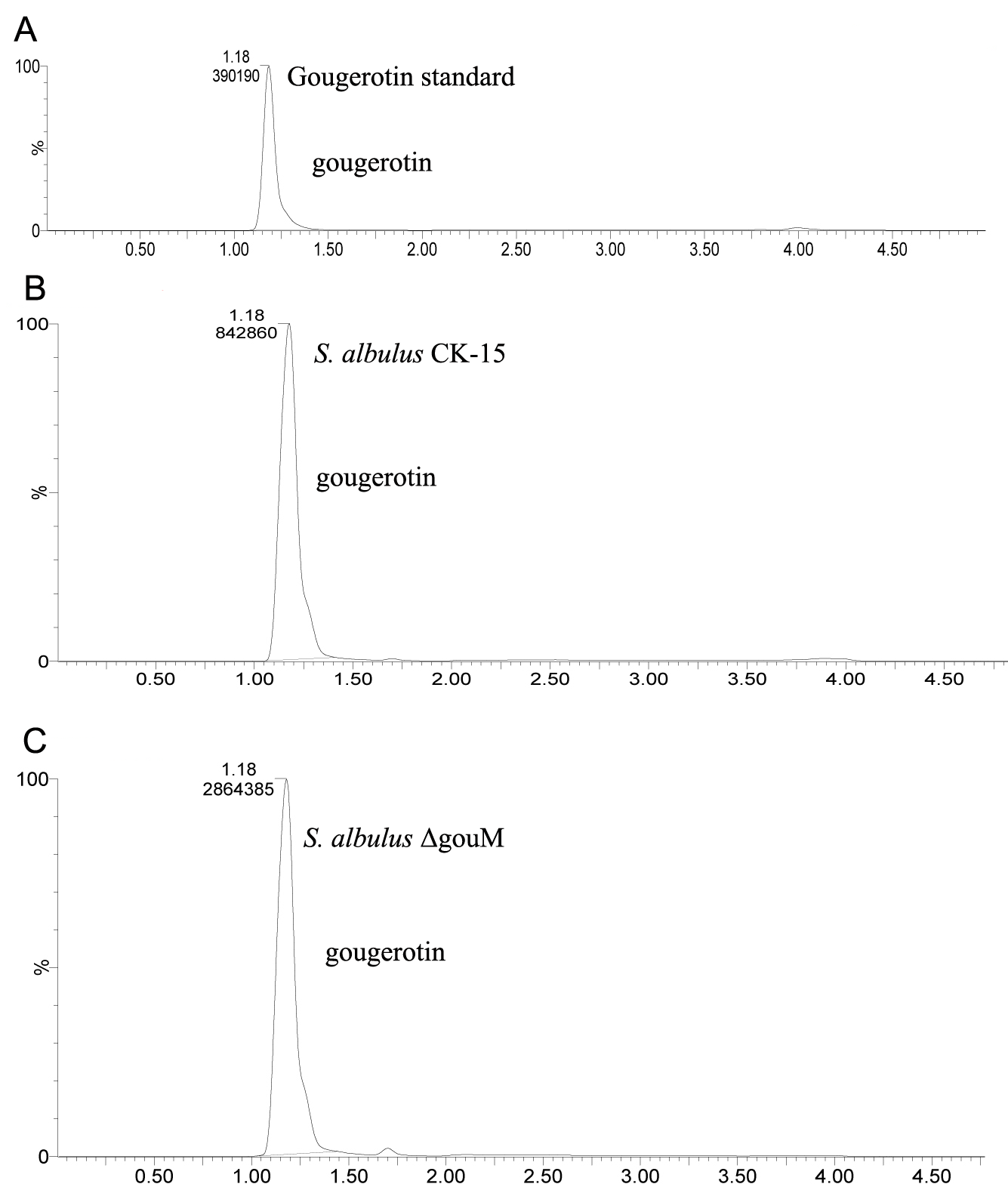
**

**Supplementary Figure 3. (A)** LC-MS analysis of standard gougerotin. **(B)** LC-MS analysis of intracellular gougerotin content in *S.albulus* CK-15. **(C)** LC-MS analysis of intracellular gougerotin content in *S. albulus* ∆gouM*.*

**
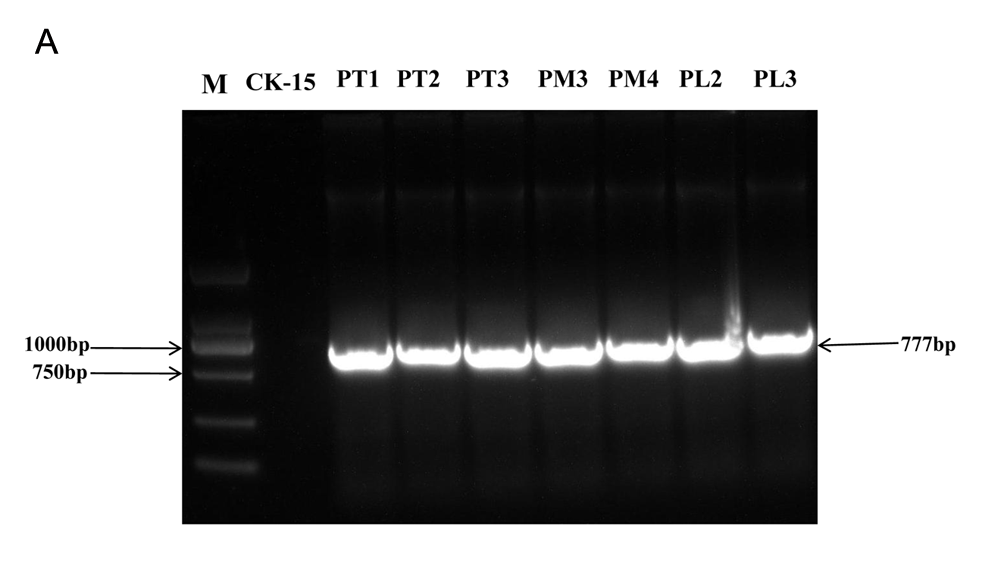
**

**Supplementary Figure 4.** Confirmation of the strains with Candidate Promoters by PCR. M, BL 2,000-bp DNA ladder; CK-15, PCR verification using *S. albulus* CK-15 genomic DNA as the template; PT1, PCR verification using *S. albulus* PT1 genomic DNA as the template; PT2, PCR verification using *S. albulus* PT2 genomic DNA as the template; PT3, PCR verification using *S. albulus* PT3 genomic DNA as the template; PM3, PCR verification using *S. albulus* PM3 genomic DNA as the template; PM4 PCR verification using *S. albulus* PM4 genomic DNA as the template; PL2, PCR verification using *S. albulus* PL2 genomic DNA as the template; PL3, PCR verification using *S. albulus* PL3 genomic DNA as the template.


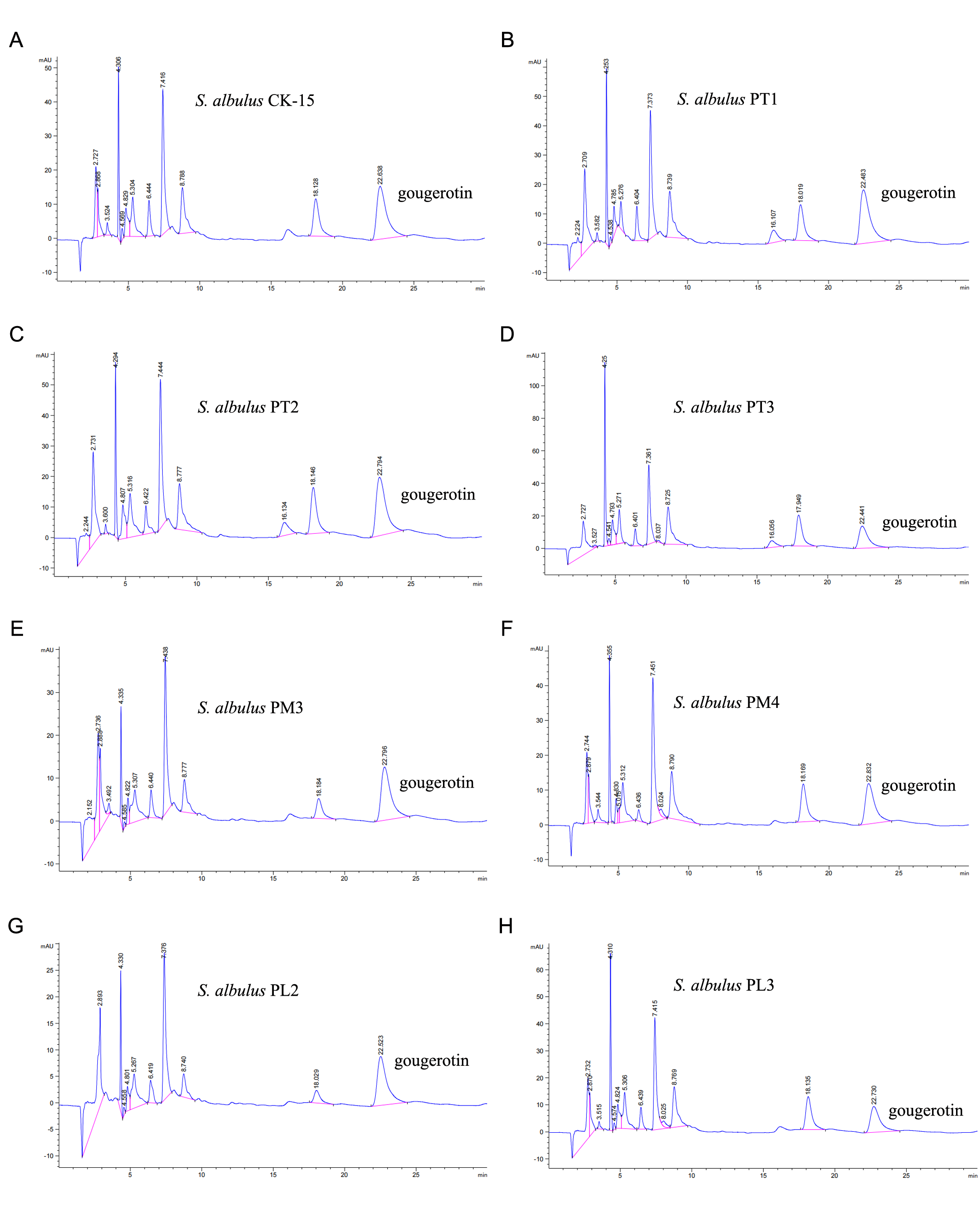


**Supplementary Figure 5. (A)** HPLC analysis of gougerotin production levels in *S. albulus* CK-15*.* **(B)** HPLC analysis of gougerotin production levels in *S. albulus* PT1*.* **(C)** HPLC analysis of gougerotin production levels in *S. albulus* PT2*.* **(D)** HPLC analysis of gougerotin production levels in *S. albulus* PT3*.* **(E)** HPLC analysis of gougerotin production levels in *S. albulus* PM3*.* **(F)** HPLC analysis of gougerotin production levels in *S. albulus* PM4*.* **(G)** HPLC analysis of gougerotin production levels in *S. albulus* PL2*.* **(H)** HPLC analysis of gougerotin production levels in *S. albulus* PL3*.*
